# Active nanoglass powers metamaterial electrocatalysis

**DOI:** 10.1093/nsr/nwag591

**Published:** 2026-09-14

**Authors:** Tao Feng, Herbert Gleiter

**Affiliations:** Herbert Gleiter Institute of Nanoscience, School of Material Science and Engineering, Nanjing University of Science and Technology, China; Herbert Gleiter Institute of Nanoscience, School of Material Science and Engineering, Nanjing University of Science and Technology, China; Institute of Nanotechnology, Karlsruhe Institute of Technology, Germany

Glasses are conventionally regarded as structurally homogeneous solids that lack the long-range periodicity of crystals. This view has been expanded by the emergence of nanoglasses, which consist of glassy domains connected by a high density of glass–glass interfaces. Because these interfaces exhibit atomic and electronic structures distinct from those of the surrounding domains, they introduce an additional structural degree of freedom for tailoring the properties of non-crystalline materials [1,2].

The functional promise of such interfaces is especially compelling in electrocatalysis. Although amorphous catalysts provide diverse coordination environments and adsorption sites, disorder alone does not ensure high activity. Catalytic configurations must also be electronically favourable, broadly distributed, and readily accessible under operating conditions. Furthermore, practical performance hinges on multiscale factors—including electrolyte transport, bubble detachment, electrical conductivity, and mechanical robustness.

In a recent paper published in *National Science Review*, Yang and co-workers tackle this challenge by combining nanoglass interface engineering with topology-engineered metallic-glass (MG) scaffolds [3]. Their catalyst (Fig. 1a) features a three-dimensionally printed MG framework (Fig. 1b) coated with a chemically heterogeneous NiMo nanoglass layer (Fig. 1c). Rather than treating the support and active phase as separate entities, the authors construct a hierarchical system in which each length scale serves a defined function: the macroscopic architecture supplies mechanical support, the interconnected channels govern liquid and gas transport, the microscale surface morphology regulates wetting and bubble adhesion (Fig. 1d–i), and the nanoscale glass–glass interfaces (Fig. 1j–l) tune the catalytic electronic states.

**Figure 1.**
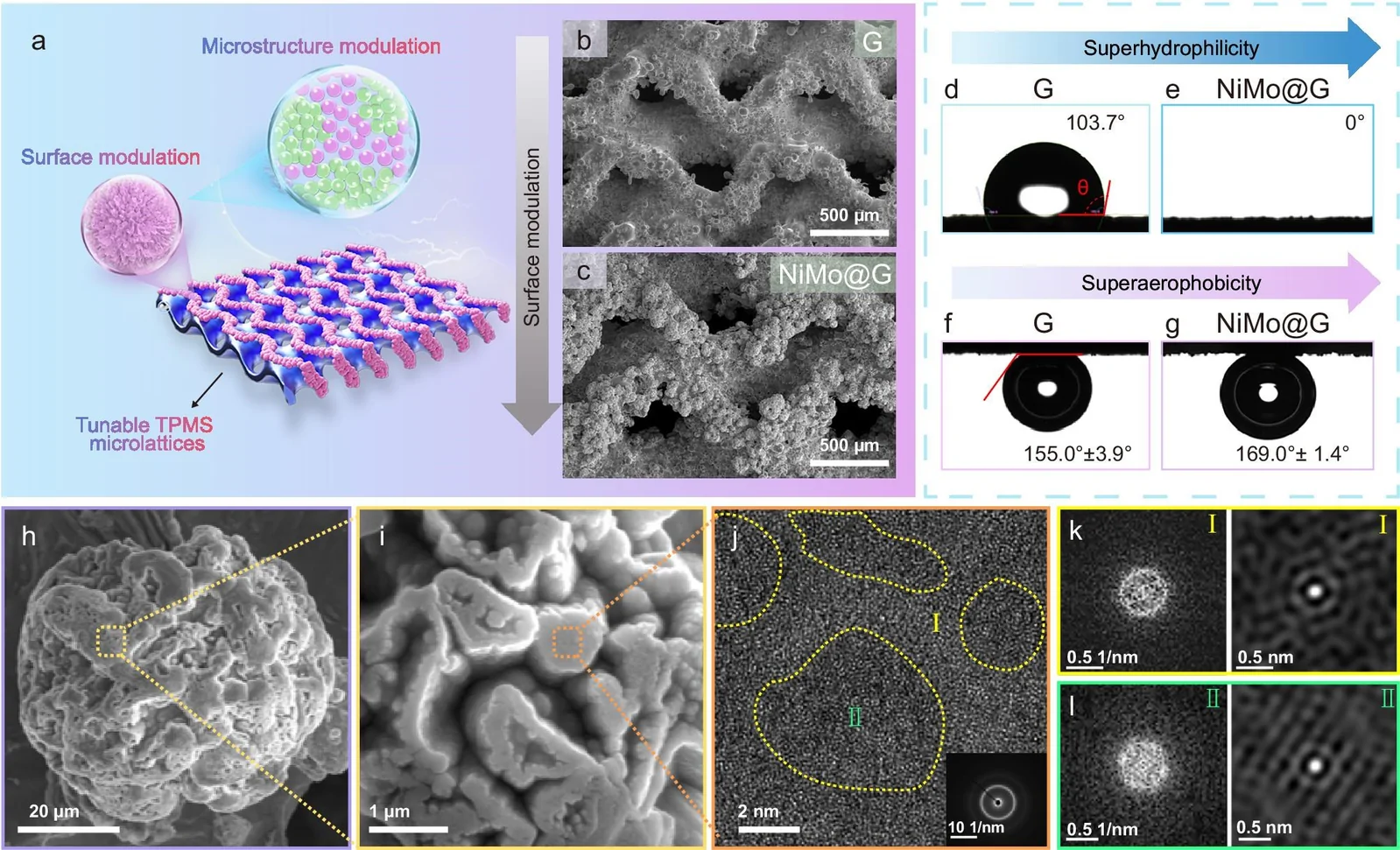
Wettability, bubble adhesion, and micro-nano structure of multiscale metamaterial catalysts. (a) Multiscale structural modulation of the integrated catalyst. (b and c) SEM images of the as-printed G-MG scaffold and the corresponding NiMo@G after PED. (d and e) Water contact angle of 103.7° on G-MG vs. superhydrophilic behaviour of NiMo@G. (f and g) Bubble contact angles in electrolyte: 155.0° ± 3.9° for untreated G-MG and 169.0° ± 1.4° for NiMo@G, indicating enhanced superaerophobicity. (h and i) SEM images of the hydrangea-like NiMo grown on the MG scaffold by PED. (j) HRTEM image with inserted SAED pattern, revealing the interfacial area (I) and clustered structure (II). (k and l) FFT and autocorrelation images of regions I and II, confirming coexisting structural inhomogeneity. Adapted with permission from Ref. [3].

In the highlighted study, electron microscopy reveals compositionally distinct Ni-rich and Mo-rich amorphous regions, approximately corresponding to Ni₇₈Mo₂₂ and Ni₆₈Mo₃₂ (Fig. 1j–l), separated by abundant glass–glass interfaces. Density functional theory calculations show that the Ni 3d-band centre at these interfaces shifts closer to the Fermi level, resulting in more favourable hydrogen adsorption and a reduced energetic requirement for the rate-determining step of oxygen evolution.

The principal advance thus lies not merely in harnessing active nanoglass interfaces, but in embedding them within a hierarchically organized catalytic metamaterial. Atomic-scale adsorption energetics are coupled with microscale wetting and bubble release, while the interconnected gyroid architecture promotes mass transport and structural stability. This strategy bridges two previously disconnected fields: metamaterial catalysts, which emphasize topology-controlled transport and mechanics [4], and amorphous catalysts, which focus on local coordination and electronic structure [5]. Its extension to multicomponent NiMoFeCo coatings further points to broad compositional tunability. Looking ahead, it remains to be verified whether the glass–glass interfaces and their associated electronic states survive prolonged water electrolysis under *operando* conditions, and whether such a hierarchical architecture can be manufactured at practically relevant scales.

By recasting glass–glass interfaces as active electronic structures and integrating them with topology-optimized MG architectures, this work converts structural disorder into a controllable design parameter. It charts a general path toward catalytic metamaterials in which atomic interfaces, surface morphology, and macroscopic transport pathways are engineered as a single, unified system.
